# Supplementary material for: Flexible and modular latent transition analysis—A tutorial using R
Source: PLoS One. 2025 Jan 17;20(1):e0317617. doi: 10.1371/journal.pone.0317617 (PMC11741568; doi:10.1371/journal.pone.0317617)
Supplement: Attachment — Submitted filename: rebuttal2-v1.docx [file pone.0317617.s001.docx]

**Rebuttal letter - revision 2**

**Reviewer #2**

I read the revised manuscript and was happy to see that it is now much clearer that the objective of the paper is to provide a tutorial for conducting LTA in R. Generally, I found that most of my previous comments were addressed well by the authors. The resulting manuscript is nearing completion, but I did have some remaining points. Nothing major, though.

***Reply:*** *Thank you so much once more for the careful reading and for the useful comments and inputs. Please see our replies below.*

• I agree with the authors that measurement invariance is and should not be a very strict requirement when doing LTA (although LTA users should be aware of it, which they not always are…) and that overreliance on significance tests to ‘establish’ measurement non-invariance is often not a good idea. However, I think that some references are needed to back this latter point up for MI-tests specifically, instead of presenting general statements about assumption tests being unsuitable in any application. This will provide future LTA users with bit more formal guidance.

***Reply:*** *Thanks for mentioning. We realized that this is a quite extensively debated topic! We now refer to the papers by Schmitt & Kuljanin and Welzel et al. who acknowledge the subjective element in the assessment of measurement invariance and downplay the role of statistical tests.*

• I would not consider the use of random starts as optional (and ten random starts strikes me as rather small number), especially when estimating more complex models. I understand that the focus of the tutorial is on the LTA part and that the importance of random starts may be assumed to be known as general LCA knowledge. However, working with model solutions at local maxima is a serious risk in LTA, so I would stress the importance of always thoroughly checking this and not only ‘when suspected’ (after all: based on what concrete clues should such suspicions arise?).

***Reply:*** *Thanks for pointing out. A good point. We've rephrased the text to stress that using multiple starting values should be done routinely. Also, we re-ran the* ***R*** *code now including the "nrep = 10" argument so that 10 random starting values were used. For wave 1 and 5 latent classes the information criteria changed a little but all other results remained unaltered (Table 1 was updated accordingly). The supplementary material on zenodo.org was also updated accordingly.*

• I previously expressed doubt about the statement that using multiple logistic regression models addresses the issue of making a proportionality assumption in traditional LTA, as I do not see how multinomial regression makes strict assumptions about this. Proportional odds are assumed in ordinal regression, but that is not what is used as a standard in LTA, as far as I’m aware. This point was not really addressed in the response or the manuscript. Maybe I misunderstand what you mean here. Could you elaborate on and/or clarify this point?

***Reply:*** *You're right that it's not a general assumption. We'd a specific multinomial model in mind but unfortunately not the general case. The text has been revised omitting such limiting but irrelevant model assumptions.*

• Although I still have some reservations about the added value of using multiple logistic regression models instead of a single multinomial regression model to calculate transition probabilities, I do feel that the authors do address this sufficiently throughout the manuscript for readers to make up their mind about this. My only remaining question then is how the authors position their approach vs. the ‘LMest’ package. They do not explicitly mention this (e.g., in the introduction), whereas this would be very informative for readers that need to decide what approach to use when they need to stick to R (I for instance like how this is usually done in J Stat Software tutorial papers).

***Reply:*** *This is a good point that we perhaps had not addressed in sufficient detail previously. From our point of view the package LMest is of limited value for analyzing more complex study designs involving hierarchical structures; indeed this was part of the initial motivation for the present work. Also, it lacks flexible handling of missing values and is limited to odds ratios as effect measures. So in the end it depends on the complexity of the LTA to be undertaken and the desired output format. We've updated the discussion accordingly.*

• By positioning their approach as a ‘3-step’ approach, the authors do raise the question how their approach relates to well-known previously described general 3-step approaches to LCA (e.g., Vermunt [2010; https://doi.org/10.1093/pan/mpq025]). For completeness, the authors may want to reflect on this.

***Reply:*** *Thanks for this very interesting paper. In principle the proposed weighted extension proposed in the paper could also be used for our approach as a means to properly propagate uncertainty from one step to the next. We now mention this great idea, which is yet another modular extension feasible using R packages, in the discussion.*

Minor remarks

• I noticed that some of the additions and changes in the text led to hard to read or grammatically suboptimal sentences (e.g., first sentence of abstract: ‘Latent transition analysis (LTA) is a useful statistical modelling approach for identifying latent classes and describe subsequent transitions between these classes over time’). I spotted similar small issues in some other parts of the text.

***Reply:*** *We've been going through the manuscript and improved a number of longer and hard-to-read sentences.*

• In the first paragraph of the introduction, a distinction is made between ‘ones that focus on characterization of determinants for belonging to certain longitudinal patterns and ones that focus on characterization of determinants of transitions between longitudinal patterns’. The latter part of this sentence is a bit confusing, given that in LTA the patterns themselves are not longitudinal, but merely the transitions between these patterns. You could consider rephrasing this slightly.

***Reply:*** *Point taken. We've revised the first paragraph somewhat to make the difference more clear.*

• You could consider adding an illustration of how to set the seed for the random number generator in Box 2.

***Reply:*** *Thanks. This is an important detail. We've included the* ***R*** *command in both Box 1 and 2.*

• In Box 3, the model contains the term ‘lca1:Age-Age’ Is this correct? I did not understand why ‘Age-Age’ rather than ‘Age’ was included here and it was not explained in the text.

***Reply:*** *Thank you for noting. The "-Age" is included to remove the use of a reference group, which is otherwise the default behaviour in* ***R****, such that estimates per group (in this latent class) are obtained directly. We've updated the supplementary material where the more detailed R-specific details are explained.*

• It might be useful to mention that the R-code to test the other covariates’ effects shown in Table 3 is given in the appendix.

***Reply:*** *Thanks. Such a sentence is now included in the manuscript.*
